# Assessing forest products usage and local residents' perception of environmental changes in peri-urban and rural mangroves of Cameroon, Central Africa

**DOI:** 10.1186/1746-4269-7-41

**Published:** 2011-12-06

**Authors:** Adolphe Nfotabong-Atheull, Ndongo Din, Léopold G Essomè Koum, Behara Satyanarayana, Nico Koedam, Farid Dahdouh-Guebas

**Affiliations:** 1 Laboratory of Systems Ecology and Resource Management, Département de Biologie des Organismes, Faculté des Sciences, Université Libre de Bruxelles-ULB, Avenue Franklin D. Roosevelt 50, CPI 169, B-1050 Brussels, Belgium; 2 The University of Douala, Faculty of Science, Department of Botany, P.O. Box 8948 Douala, Cameroon; 3 University of Yaoundé I, Faculty of Science, Department of Plant Biology, P.O. Box 812 Yaoundé, Cameroon; 4 Laboratory of Plant Biology and Nature Management, Mangrove Management Group, Vrije Universiteit Brussel-VUB, Pleinlaan 2, B-1050 Brussels, Belgium; 5 Institute of Oceanography, Universiti Malaysia Terengganu - UMT, 21030 Kuala Terengganu, Malaysia

## Abstract

**Background:**

Deforestation is one of the most ubiquitous forms of land degradation worldwide. Although remote sensing and aerial photographs can supply valuable information on land/use cover changes, they may not regularly be available for some tropical coasts (*e.g*., Cameroon estuary) where cloud cover is frequent. With respect to mangroves, researchers are now employing local knowledge as an alternative means of understanding forest disturbances. This paper was primarily aimed at assessing the mangrove forest products usage, along with the local people's perceptions on environmental changes, between Littoral (Cameroon estuary) and Southern (mouth of the Nyong River and Mpalla village) regions of Cameroon.

**Methods:**

The data from both locations were obtained through conducting household interviews and field observations.

**Results:**

In the Cameroon estuary (Littoral region), 69.23% of respondents (mostly elders) could distinguish two to four mangrove plants, whereas the informants (65.45%) in the mouth of the Nyong River and Mpalla village (mostly young people interviewed from the Southern region) are familiar with only one or two commonly found mangroves. Also, more respondents from the Cameroon estuary are depending on mangroves for fuelwood (*Rhizophora *spp.) and housing (*Rhizophora *spp., *Avicennia germinans *(L.) Stearn and *Nypa fruticans *(Thumb.) Wurmb.) purposes, in contrast to Nyong River mouth and Mpalla village. Although local people perceived wood extraction as a greater disruptive factor, there are several causes for mangrove depletion in the Cameroon estuary. Among others, over-harvesting, clear-felled corridors, sand extraction and housing were found important. Furthermore, a decline in mangrove fauna composition (in terms of fishery products) was recorded in the Littoral as well as Southern regions. However, the causes of such perceived negative changes were not similar in both cases.

**Conclusions:**

Findings of this study highlight the need to improve sustainable management of the mangrove ecosystems through afforestation (in large impacted areas), selective removal of senescent tree stems and branches (in little damage stands), regulating sand extraction and housing activities, and creating awareness and law enforcement.

## Background

Deforestation is a major environmental concern that societies around the world are currently facing [1-4]. One of the most obvious cases pertains to mangrove forests that are found along the tropical and subtropical coastlines. Mangroves are disappearing at a rate greater than or equal to adjacent terra-firme rain forests [5]. The causes of loss have been mainly attributed to anthropogenic activities [6-8]. Indeed, mangrove wood products remain an important source of building and fuelwood material for coastal communities [9-14]. Therefore, in order to ensure that future generations enjoy the ecosystem services provided by mangroves [15,16], it is fundamental to assess the environmental changes therein.

Though remote sensing and aerial photographs can supply valuable long-term data on forest cover changes, they are not always available for some parts of the tropics (*e.g*., Cameroon coast in Central Africa) where cloud cover is frequent. With a scarcity of such datasets in the developing countries, several researchers are now employing local knowledge as an alternative means of reconstructing changes in the mangrove forests [17,4,19]. The consistency of this alternative approach is related to the fact that local inhabitants can provide insight into certain environmental changes which may be missed by exact sciences alone [20-22]. Hence, local people's perception on long-term changes in the mangroves is of great importance [23].

In this study, residents of the Littoral and Southern region of Cameroon were surveyed for their forest products utilization and perception on mangrove cover changes. The aims were: (a) to confront the usage of mangrove wood products in the Southern Cameroon (close to the mouth of the Nyong River and Mpalla village (Kribi)) and the Littoral (Cameroon estuary) regions; and (b) to assess local resident's perceptions on environmental changes in the two regions.

## Methods

### Study area

**Figure 1:**
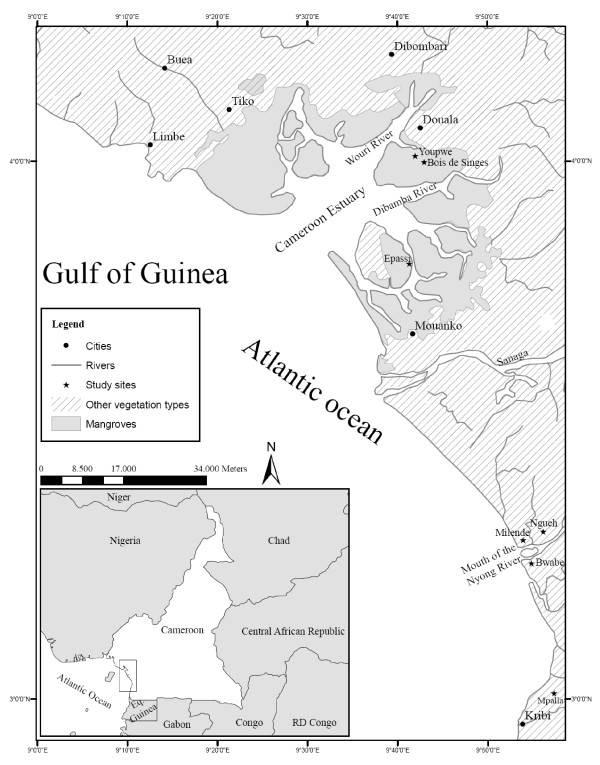
**Map showing the location of study area and the seven selected sites**.

Mangroves in the mouth of the Nyong River (3° 15' N and 9° 55' E) and Mpalla village (2° 59' N and 9° 55' E) are bordered by extensive terrestrial (evergreen) forest (Figure 1). These coastal ecosystems annually receive about 2870 mm of precipitation. The Cameroon estuary (3° 53' N and 9° 38' E), with its two irregular seasons and a mean annual rainfall of 3988 mm, shares more than 30% of its continental limit with Douala, the first most populated city in Cameroon [24]. The Littoral and Southern regions are also distinguishable in terms of mangrove area (small patches in the mouth of the Nyong River and Mpalla village and large units in the Cameroon estuary) and anthropogenic disturbances (rural in Nyong and Mpalla village and peri-urban in the Cameroon estuary), and therefore makes this study highly relevant for appropriate conservation and management practices.

### Data collection

The data were generated through conducting semi-structured interviews with local residents (mostly fishermen living in the immediate vicinity of mangroves) and our field-based observations. Between 15 December 2007 and 25 February 2008, a semi-structured questionnaire (based on [10,25]) was used to investigate the two peripheral quarters of Douala (*i.e*., Youpwe and Bois de Singes), two large villages (Epassi and Mpalla), and three small neighbouring villages (Milende, Ngueh and Bwabe) (Figure 1). The number of respondents was 25, 16, 24, 30, 12, 6 and 7 at Youpwe, Bois de Singes, Epassi, Mpalla, Milende, Ngueh and Bwabe, respectively. Most interviews were done in French, except in Mpalla and Bwabe where a local language called 'Batanga' was used with the help of translator. Household visits were carried out at the end of the day after locals came back from their occupation/fishing activities. The households were sampled opportunistically (*i.e*., respondents encountered in the houses were interviewed whereas people away from their homes were not questioned). Nine homes were hostile to our visit and five people refused to answer.

In total, 120 questionnaires were carried out in this survey. Amongst these, 110 (60 in the Cameroon estuary and 50 at the mouth of the Nyong River and Mpalla village) were used for statistical analysis and 10 were discarded because of incoherent information. To avoid recurrent information, only one person (> 20 years of age) per house was questioned. The preference was given to elderly people who were living in the site for more than a generation and therefore could relate changes that occurred in adjacent mangroves to certain events or conditions. Interviewees were asked if the surrounding mangrove forest had increased, decreased or remained unchanged in cover. Likewise, an attempt was made to distinguish whether local informants perceived mangrove forests as less or more degraded over time. They were urged to provide the causes of the reported changes along with its consequences in their livelihood. Local inhabitants were also asked questions about any observed disturbances in the mangrove ecosystem.

A catalogue showing mangrove plants and their physiognomy, leaves, fruits and flowers was used (as described in [10,25]) to assess the level of each respondent's knowledge on mangrove species. Since most of the informants were unable to distinguish the characteristic differences between *Rhizophora racemosa *Meyer- the dominant species in Cameroon mangrove forests (*cf*. [24,13]), *R*. *harrisonii *Leechman and *R*. *mangle *L. (classification based on [26]), we have recorded only its genus name when they referred to any one of the above three species for use.

Data on local people's demography (*e.g*., age, gender, etc), along with plant vernacular names and their parts used for timber and non-timber forest products including ethnopharmacological (*i.e*., human diseases treated, methods of remedy preparation and administration) and chemical (*e.g*., dye preparation) usages, were gathered from the household interviews.

### Data analysis

Chi-square tests (χ^2^) (SPSS *v*. 16.0) were used: (a) to determine if there was any significant difference (p < 0.05) in mangrove use by region (*i.e.*, Littoral and Southern), and (b) to confront the level of degradation reported by informants in the two regions. Principal component analysis (PCA) (a routine implemented in PRIMER *v*.6) was performed to assess the patterns of usage perceived as largely responsible for mangrove degradation in each region. It is important to highlight that the answers on the ethnobotanic (traditional species usage) section of the questionnaire reported by those people who could not even distinguish single species were not considered, whereas their general perceptions on environmental changes were taken into account.

## Results

### Local people's knowledge on mangroves

The majority of the informants were males and their age ranged between 21 and 72 years (45.09 ± 12.16; mean ± 1SD). In both parts, respondents referred to the mangrove forest as 'matanda', and identified *Rhizophora *tree as 'itanda' (Table 1). In the Cameroon estuary, 69.23% of respondents (mostly elders) could distinguish two to four mangrove plants, whereas the informants (65.45%) in the mouth of the Nyong River and the Mpalla village (mostly young people) are familiar with only one or two commonly found mangroves (*e.g*., *Rhizophora *spp. and *Avicennia germinans *(L.) Stearn).

**Table 1:** Subsistence uses of mangrove woods in Littoral and South Cameroon.

Mangrove taxa	Local name ('batanga', 'Duala'*)*	Part used	Uses
*Rhizophora *spp.	'itanda'	stems	commercial firewood and charcoal, precarious house construction (poles), plank-making, flagstone support, fabrication of tables, chairs, boats/canoes immobilisation.
		branch	domestic firewood, fencing.
		bark	malaria treatment (external usages), stop ping of external haemorrhages, stomach illness (ingurgitation), tooth decay treatment, tainted fishing net.
		root	fabrication of shuttle.
*Avicennia germinans*	none reported	stems	commercial planks for construction, paddles, traditional boat/canoe construction, bench, chairs.
		branch	domestic firewood.
		leaves, bark	malaria treatment (external usages), smelly/stinky body.
*Laguncularia racemosa*	none reported	stems	Boat/canoe construction, paddles, firewood (household consumption).
		leaves, bark	treatment of measles, gonorrhoea, malaria, stomach illness.
*Nypa fruticans*	'Lende la djengu'	leaves, fruits	mat confection, wall dressings. house roofs decoration.

### Utilization of mangrove resources

During the field observation, a total number of five mangrove species namely, *R. racemosa, R*. *harrisonii*, *A*. *germinans*, *Laguncularia racemosa *(L.) Gaertn. f. and *Nypa fruticans *(Thumb.) Wurmb. were found in the Littoral region of Cameroon, and the same is true for the Southern region except that *R. mangle *replaced *R*. *harrisonii*.

**Figure 2:**
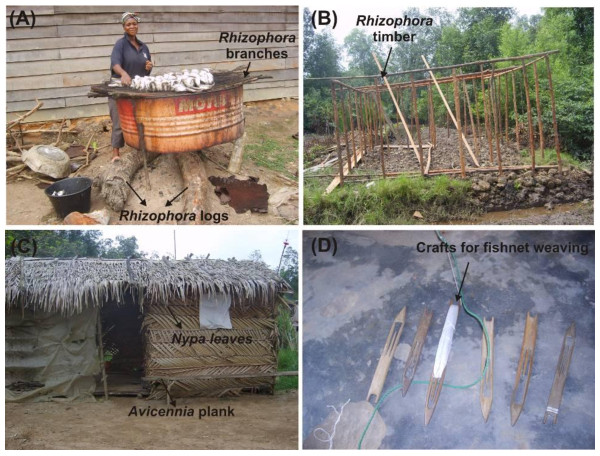
**Photographs illustrating different mangrove uses in Cameroon**: (A) Mangrove poles obtained from *Rhizophora *spp. are used for fish smoking ready to sell in the local market (Youpwe). (B) Use of *Rhizophora *small timber for precarious house building at Youpwe. (C) *N*. *fruticans *leaves used for walls covering at Epassi. (D) 'Shuttle' crafted from *Rhizophora *prop roots at Mpalla village (Kribi). (Photographs by ANA).

In Cameroon, the mangrove products are used as fuelwood (*e.g*., charcoal making, cooking or heating of the traditional henhouses), construction materials, service wood, dye or medicine (Table 1). Within the Littoral region, the use of mangroves for fuelwood (Figure 2A), construction materials (Figure 2B) or service wood (Figure 2C, D) in Epassi is almost similar to Youpwe (χ^2 ^= 0.82; d.f. = 2; p = 0.13). With regard to the three aforementioned uses, mangrove wood collection is not significantly different in Bois de Singes and Youpwe (χ^2 ^= 5.18; d.f. = 2; p = 0.06). In contrast to Epassi, only few residents were using mangrove either as fuelwood, construction materials or service wood in Bois de Singes (χ^2 ^= 6.87; d.f. = 2; p = 0.009) (Table 2). In the Southern region, mangrove harvesting for the above purposes did not differ much between Mpalla and the mouth of the Nyong River (Milende, Ngueh and Bwabe) (χ^2 ^= 0.97; d.f. = 2; p > 0.05).

**Table 2:** Mangrove wood products used in different sites. Comparisons in mangrove uses for fuelwood, house construction and service wood are made within and between the two regional sites.

Sites	# Question-naires	Mangrove uses				
		
		Fuelwood	Construction	Service wood	Medicinal	Chemical
**LITTORAL REGION**						
**Cameroon estuary**						
Youpwe	20	16	10	6	0	0
Bois de Singes	16	6*	0*	0*	0	0
Epassi	24	**24**	**16**	**15**	3	3
**Total**	60	46*	26*	21*	3	3
**SOUTHERN REGION**						
**Mouth of Nyong River**						
Milende	12	6	1	3	3	1
Ngueh	6	0	0	2	1	0
Bwabe	7	2	1	0	4	0
**Kribi**						
Mpalla	25	8	2	10	0	3
**Total**	50	**16**	**4**	**15**	8	4

Areas showing significant difference in mangrove uses are represented in bold with similar font colours. Values represent the number of respondents indicating that they use mangrove plants (n = 110).

* p < 0.05.

Overall, the collection of mangrove wood products was found more important in the Cameroon estuary than in the mouth of the Nyong River and Mpalla village (χ^2 ^= 6.75; d.f. = 2; p < 0.05) (Table 2). *Rhizophora *timber was used for house construction, especially in the Cameroon estuary (Table 1) (Figure 2B). The wall dressings and planks were obtained from *Rhizophora *spp. and *A*. *germinans *trunks. *N*. *fruticans *leaves were also used for wall dressings. In order to reduce the entry of air currents inside the precarious houses (in Epassi village), the walls are first covered by plastic sheets and then by *N*. *fruticans *leaves (Figure 2B, C). *Rhizophora *trunks constituted an important component of traditional chairs, benches and tables, while *A*. *germinans *stems are used for canoe construction and paddle fabrication. The latter two wood products were also obtained from *L*. *racemosa *trunks.

There were no significant differences between the two regions in terms of mangrove usage for medicine and/or dye for clothes or nets (χ^2 ^= 0.46; d.f. = 1; p > 0.05) (Table 2). The collection and transformation of mangrove plants into medicinal products is usually done by women. However, mangrove medicine is popular and in use only at the wedged zones like Epassi, Milende, Bwabe and Ngueh. *A*. *germinans *leaves and bark were used to treat malaria patients. In this context, the bowl or pot with boiled water and *A*. *germinans *leaves/bark, together with the sick person, are covered by a thick blanket for inhaling. The same technique was also applied to cure measles and gonorrhoea, but with the leaves and bark of *L*. *racemosa*. According to local people, high perspiration could even eliminate bad odour from the patient's body. The decoction after boiling *Rhizophora *bark was used to stop external haemorrhages and to cure tooth decay. The usage of mangrove chemicals was particularly reported from Mpalla, Epassi and Milende. The liquid obtained from fresh *Rhizophora *bark is used to dye and preserve faded clothes and cotton fishing nets, the latter of which was also reported to attract more fish.

The tool 'shuttle' for weaving fishnets is locally made from *Rhizophora *prop roots (Figure 2D). They are crafted by artisans who were not always fishermen (Figure 2D). The shuttle's central part, which was empty except for a thin axis, was used to spin around fishing thread, and the entire tool was then used with the concave extremity to weave the fishing net. These shuttles are also available in the local market at Mpalla village with a price ranging from 0.30 to 0.45 EUR (0.35 ± 0.05 EUR) (the exchange rate at the time of data collection was 1 EUR = 655.95 FCFA).

### Environmental changes in mangroves

#### Perceived changes to mangrove stands

According to local people, the mangrove forests in Mpalla (Kribi) and in the mouth of Nyong River were not as severely degraded as in the Cameroon estuary (χ^2 ^= 67.94; d.f. = 2; p < 0.001) (Table 3). Here, more than half of the interviewees (66.15%) reported considerable damage to mangroves (Table 3). On one hand, they recounted the loss of mangroves in some areas due to anthropogenic activities, and on the other hand they referred to natural degradation of the remaining stands. About a third of the population (32.31%) supported that mangroves were not threatened and therefore remain unchanged, whereas a small minority (1.54%) perceived mangrove degradation only slightly.

**Table 3:** Local residents' perception of changes occurred in mangroves (n = 120).

Sites	Number of respondents	Reported level of degradation		
		
		No change	Little	Large
**Cameroon estuary**	65	21 (32.31%)	1 (1.54%)	43** (66.15%)
**Mouth of Nyong River and Mpalla village**	55	23 (41.82%)	31 (56.36%)	1 (1.82%)

** p < 0.001. Values in parentheses indicate percentage of the respondents per type of perception per site.

**Figure 3:**
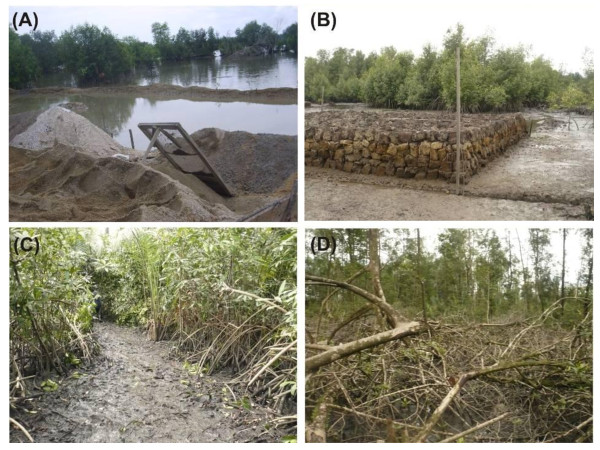
**Mangrove land degradation in Cameroon**: (A) Sand extraction within mangrove forest. The background shows uncut sparse adults trees. (B) Elevated areas of previously cleared mangrove stands waiting for housing. (C) Clear-felled corridor within the mangrove used for wood transport or as access path. (D) Complex of cut branches and prop roots (*Rhizophora *spp.) left in situ. (Photographs by ANA).

In the Cameroon estuary, several informants perceived mangrove wood extraction as a major disruptive factor. They all agreed to the fact that their own houses were constructed in the place(s) of mangroves. An important part of these participants ranked housing activity as a second threat. Though all respondents generally agreed with the ecological importance of mangrove (*e.g*., coast stabilization), they declared that this ecosystem is the only place where they could go for clear felling and free housing. Besides this assertion, other residents reported that the daily dumping of untreated domestic wastes could contribute to mangrove degradation locally. However, significant evidence of forest degradation was not observed in and around the concerned areas by the authors. Respondents had an opinion of less potential damage to mangroves with sand extraction (Figure 3A) since they disturb only substratum. It is also important to highlight that the areas (up to a distance of 3 km) reported with municipal and domestic pollution are free from sand extraction activities.

In the mouth of the Nyong River and the Mpalla village, about 56.36% of respondents indicated that the existing mangroves were less impacted (Table 3). At the same time, a few residents from Mpalla explained that the fringe mangroves (bordering the sea) have been cleared by the foreign residents for land development projects. More than one-third (41.82%) of the Southern informants still argue that the mangrove was less disturbed, while some residents of the latter category admitted that they occasionally cut mangrove trees growing in the immediate vicinity of their houses. In contrast, several fishermen in Mpalla village indicated that they were no longer able to reach to some of their old fishing grounds due to extensive growth of *Rhizophora *spp. prop roots and mud deposition along the channels. Indeed, people of this region clearly stated that they often prefer non-mangrove species (*e*.*g*., *Lophira alata *C.F. Gaertn. locally called as 'bojambi' or 'azobé''), with nearly the same calorific value as *Rhizophora *spp., for fuelwood.

The other non-reported, but field-observed, anthropogenic activities such as digging, landfill, dyke construction and large clear-felling would also contribute to mangrove degradation. In this case, the mangrove trees are uprooted and then filled with sediment (brought from nearby areas) to make those places suitable for housing (Figure 3B). Interviewees claim that these elevated areas could also protect their homes from (daily) tidal inundation. Another notable anthropogenic factor observed in Youpwe and Epassi, but not stated by informants, was the clear-felling of young mangrove trees (in 1.5 m width as a corridor) from inside the forest to the water channel(s) where they anchor boats (Figure 3C). In order to facilitate wood transportation, big logs were progressively pushed on mud along the clear-felled corridors. These corridors are also used as pathways later on by several loggers and local people. Unlike large stems of *Rhizophora *which were cut, split and transported for commercial purposes, their branches and stumps (with under-ground roots) are usually left in the field (Figure 3D).

**Figure 4:**
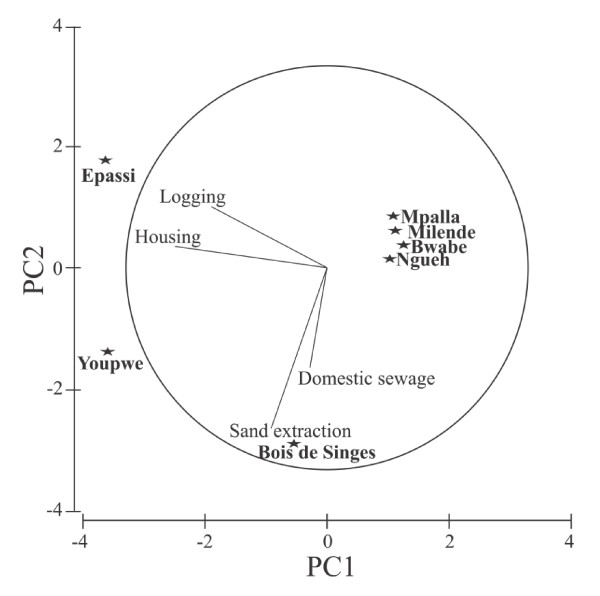
**Principal Component Analysis of the perceived causes of mangrove degradation**. It can be observed that each sample site in the Cameroon estuary (*i.e*., Epassi, Youpwe and Bois de Singes) is located close to its major degradation threat perceived. Nevertheless, the four sites on the top right corner (Mpalla, Milende, Bwabe and Ngueh) are seen less influenced by those four recognized mangrove threatening factors. Each star indicates the position of its respective sampling site in the PCA plane with reference to the perceived causes of mangrove degradation. The length of each vector line represents the importance of that particular variable's contribution to the PCA axis. The circle represents the graphical illustration of the correlation between the variables and the principal components. (This analysis does not take into account data obtained from field observation).

The PCA with perceived causes of mangrove degradation in each site explained a total variability of 71.30% on axis-1 (*i*.*e*., eigenvalue of 7.03), and 26.30% on axis-2 (*i*.*e*., eigenvalue of 2.59) (Figure 4). The linear coefficient of original variables (perceived causes) making up PCA revealed that the respondents in Epassi saw logging and housing (clustered on negative and positive sides of the both axes) as the main causes of mangrove degradation (Figure 4). In contrast, Bois de Singes was characteristic of the mangrove damages from municipal/domestic sewage pollution and sand extraction (clustered on lower parts of the axes). The informants at Youpwe site also perceived mangrove degradation as a consequence of housing and sand extraction (standing in-between Epassi and Bois de Singes). The Southern villages were all characterized by few concerns of degradation in mangroves, and clustered along the upper parts of the two principal components (*i.e*., PCA 1 and 2).

#### Perceived changes in faunal composition of mangroves

Although there were few logging activities in the mouth of the Nyong River, local people unanimously shared the opinion that trawler activities along the shore resulted in a decline in local fish catch inside the mangroves. This negative change was reinforced by the increased number of local fishermen. A decrease in the population of silurid, tilapia, mullets, shrimp, oyster and crab species, as stated by informants in the Mpalla village, was probably related to changes (*e.g*., siltation, reduced water circulation, etc) that occurred in the waterways. Though such decrease in mangrove faunal composition was recorded in the Littoral region, many interviewees linked it to the anthropogenic disturbances prevailing (*e.g*., harvesting, clear-felling, domestic sewage, pollution and sand extraction).

## Discussion

### Mangrove uses

Almost in every tropical and sub-tropical regions of the world, mangrove forests are widely harvested for different purposes [6-8,10,13,24,27,28]. In the Cameroon coastal regions, we found that stems obtained from *Rhizophora *spp. and *A. germinans *are commercially important whereas their branches, in a few cases, were preferred for domestic utilization. The choice here for making those houses with small *Rhizophora *trees was based on its aesthetic value, availability, easiness to harvest and rot-resistance characteristics. Studies have documented that different mangrove species have different wood properties, making some more suitable than others for specific uses [8]. The stems of *Rhizophora*, characterized by its hard nature and richness in tannins, were perceived to be highly marketable goods for their potential use as firewood, charcoal and construction materials [9]. This wood is less appropriate for plank-making than that of *A. germinans*, the latter being easier to split. Except in Epassi village (located 25 km away from Douala city), none of these two species were used for 'banda' construction (a table-like structure made from mangrove timber to smoke fish) as recorded previously by Nfotabong Atheull *et al*. [13] in the Douala-Edea reserve. However, instead of 'banda', the locals in Youpwe are using hollow iron drums (with its upper surface covered by *Rhizophora *branches) for fish smoking. The advantage with this kind of oven is said to be for keeping the smoked fish warm for longer durations.

Although there were no reports on the use of *Rhizophora *prop roots for 'shuttle' fabrication in the Littoral region, this is an activity carried out by some fishermen in Mpalla village. The strength and durability of the 'shuttle' reduced the frequency of prop root harvesting (about two prop roots per adult tree are cut) and tree felling. In addition, some artisans stated that their frequency of visits to the mangrove was limited because of the harsh environment. While both leaves and bark of *A. germinans *and *L. racemosa *are used in ethnopharmaceutical purposes, only that of *Rhizophora *bark serves medicinal and chemical purposes widely (Table 1). Similar uses of *A. germinans*, *L. racemosa *and *Rhizophora *have been reported by Kovacs [17] and Hernández-Cornejo *et al*. [6]. The bark of the above species is an excellent remedy for curing several diseases (malaria, measles and gonorrhoea) because of its rich soluble tannins [29] which may inhibit microbial activity [30]. Similar chemical usage (for dye preparation) of the barks obtained from *Rhizophora *and *Ceriops decandra *(Griff.) Ding Hou was also reported from Coringa mangroves in the Godavari Delta, India [25].

### Perceived environmental changes in mangroves

Local people settled within or around the mangroves forests can provide information that contribute to the identification and/or evaluation of factors inducing environmental disturbances in mangrove forests (see [4,11]) since their observations span over a long-term [31]. Their perceived degree of mangrove damage may vary depending on location and place of the mangrove stands studied. For instance, the local residents' perceptions on environmental changes differ between the Littoral and Southern parts of the Cameroon coastlines (see results).

#### Mangrove changes in the Littoral region

**Figure 5:**
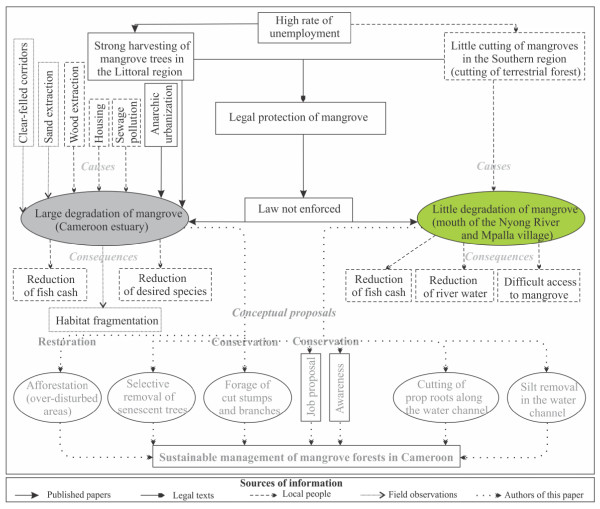
**Flow chart showing the causes of mangrove degradation as well as remedies (conceptual proposals) for its proper conservation and management strategies in Cameroon**. Each source of information is represented by different types of arrows. The causes as well as consequences of large (Littoral region) or little (Southern region) degradation of mangrove forests are also differentiated.

In the case of Cameroon estuary, high rate of unemployment coupled with lax government enforcement and the peri-urban situation of mangroves favoured the large degradation of forests (Figure 5). The local loggers, while waiting for a formal job, often cut mangrove wood for commercial utilization [13]. As mentioned in the results, the majority of local people perceived that wood harvesting and house construction are significant causes of mangrove damage. Instead of focusing on both causative factors, it is always worth to highlight the combination of issues which are truly responsible for changes in the mangrove forests (Figure 5).

Another possible cause for extensive mangrove degradation in the Cameroon estuary is population growth, which can uplift the anthropogenic pressure on adjacent ecosystems [24,32,33]. This threat is of great concern since a considerable proportion of informants living in the Littoral region stated that they can liberally use mangrove areas for housing.

The local residents recognized that some mangrove stands were already replaced by houses. They further explained that house construction often starts with dyke enclosures (to reduce water supply), then mud cracking and finally tree clear-felling. With regard to these statements and the findings of Din and Blasco [34], anarchic urbanization constitutes an additional significant threat to mangrove degradation in the Cameroon estuary (Figure 5). The extent of subsequent damage depends on the intensity, persistence and periodicity of forest disturbance [35,36], as well as the type of anthropogenic activity carried out. Likewise, cutting could also lead to a dramatic effect from which recovery may be slow or even impossible [8,12,16,28,35]. The speed of recovery is function of the forest type affected and the density of seedlings and established propagules prior to cutting. For instance, in disturbed *Rhizophora *zones, cut branches and prop roots left in the site, often trap mangrove propagules and prevent their dispersal into the inner disturbed zones. However, this phenomenon is less pronounced within the disturbed monospecific stands of *A. germinans *where its seed retention by cut branches and pencil roots is limited.

Contrary to informant information, the impact of untreated anthropogenic sewage pollution on mangroves cannot be proved with the current observations. In addition, some studies have indicated an increase of mangrove [37,38] and shrimp [39] growth, with no apparent negative effects [23,40,41].

A detailed analysis of the PCA has shown that the perceived causes of mangrove damage differ across the sample sites. In the Cameroon estuary, the site separation is probably due to higher heterogeneity amongst the sample sites with respect to some patterns of use. For example, people from the Bois de Singes are only concerned about municipal/domestic sewage pollution. Such kind of pollution is common in mangroves of East Africa [23]. Residents of the aforementioned site, like those of Youpwe, also qualified sand extraction as a threat to mangrove. But Youpwe residents attributed a similar potential threat to sand extraction and housing. Likewise, sand extraction and housing were rated almost equitably in Epassi. Therefore, the discrepancies amongst sites likely denote the differences in site locations. For instance, in a non-urban site like Epassi, sand extraction and municipal/domestic sewage pollution are unknown activities and accordingly weighted more the perceived negative effects of logging and housing. However, in the peri-urban site such as Youpwe, the diverse perceived threats could explain the intermediate position of this site. Based on our field observations, clear-felled corridors in the mangrove areas, sand extraction, urban expansion and housing and associated activities (dyke construction, large clear-felling and digging) could be seen as major threats creating almost irreversible changes in the species composition and distribution. Of course, it is clear from the results that clear-felled corridors could lead to the fragmentation of mangrove habitats in Youpwe and Epassi. Moreover, in some mangrove stands at Bois de Singes, the destruction of propagules by sand extraction might prevent natural regeneration, which could be masked by the continued presence of patched trees (see Figure 3A). This was similar to what Lewis III [42] observed and quoted it as 'propagule limitation'. Notice that the concerns are not restricting to the propagules alone since residents in the Littoral region recognized low fish production (including gastropod, bivalves and crabs) with high anthropogenic disturbances.

#### Mangrove changes in the Southern region

Similar to the Littoral region, there is also high unemployment rate and no enforcement of laws protecting mangroves in the Southern region. Yet, mangroves in the mouth of Nyong River and Mpalla village were seen degraded only slightly (Figure 5). This is clearly illustrated in the PCA by a grouping of sites sampled in the Southern region. The low perceived threat to mangroves might be due to less dependence of residents on this intertidal forest. In fact, the Mpalla and other villages in the mouth of the Nyong River are surrounded by terrestrial rain forest from which the local people obtain timber and non-timber forest products (Figure 5).

In the locations where there is an abundant supply of hardwood timber from other forest types (*e.g*., Australia), the use of mangrove wood has been very limited and several mangrove forests remain well conserved [7]. In the Mpalla village, this kind of situation must be responsible for rapid growth of *Rhizophora *prop roots and siltation (reducing the width and depth of water channels), and ultimately less access to some traditional fishing grounds. This is in strong opposition with the mangrove of Nziou (Kribi located, 3 km from Mpalla) which has been deforested for land development projects (pers. obs.). However, the reported changes in Mpalla village also have a natural meaning, as it is the only water channel carrying seawater into mangrove (the small creek mouth) and further upstream with narrowing width. Interestingly, the fishermen have also learnt to manage this phenomenon. Indeed, they dam-up the small creek mouth in November (beginning of dry season) and open it in December (mid dry season) to catch the fish that were seeking refuge or that have been trapped in the mangrove and try to migrate back to the ocean.

### Mangrove restoration and conservation

Overall, the local communities are aware of changes that occurred in their adjacent mangrove forests and most of them were willing to abandon mangrove cutting provided they find alternatives. In this context, development of rural commercial farming as one of the possible alternate jobs to mangrove cutting might reduce anthropogenic pressure on the Cameroon mangrove forests. On one hand, by diminishing the rural exodus from villages to Douala city, human pressure on the peri-urban mangrove resources may be reduced. One the order hand, as stated by Linares [43], many local people currently degrading and destroying the peri-urban mangrove ecosystems could return to their original communities to develop farming activities. In addition, better access to education and credit (a scheme designed to promote individual entrepreneurship) along with the adoption of specialized fisheries might reduce the extraction of fuelwood and clearing for house construction [44]. Moreover, the development of ecotourism inside the mangrove forests might imply a progressive livelihood diversification away from the traditional overexploitation of these intertidal forests.

For the Cameroon estuary, the restoration or rehabilitation might only be considered as a priority when mangrove forests have been cut to such an extent that it could no longer self-correct or self-renew (*cf*. [45]). Under such conditions, an afforestation policy for the mangrove ecosystem involving its reproduction and growth must be undertaken [46]. In this case, reforestation projects should take into account the soil characteristics (*cf*. [47]) and seedling availability. However, in the zones under less harvest pressure, efforts should only emphasize on selective removal of senescent tree stems and branches, natural regeneration and awareness of local residents on the importance of mangrove ecosystems (Figure 5). Local people should look for stumps and cut down branches previously left in the field and thereby reduce levels of cutting. Considering the fact that changes in hydrology could impact mangroves at some distance, causing the gradual die-back of particular species or entire stands [48], expanded *Rhizophora *prop roots and accumulated mud in the waterways at Mpalla (Southern region) should be regulated through selective cutting and digging processes (for silt removal in the channel).

## Conclusions

This survey provided details on the importance of Cameroon mangrove forests in local livelihoods, along with the people's perceptions on environmental changes that have occurred in these coastal habitats. Both large and little damage to existing mangrove stands in the Littoral and Southern region of Cameroon were clearly identified through both questionnaire and field-based observations. It is a complex mix of causes (*i.e*., over-harvesting, housing, clear-felled corridors and sand extraction) that have led to environmental changes in the mangroves of Cameroon estuary. In contrast, the little damage of mangrove forests around the mouth of Nyong River and Mpalla village (Kribi) is due to small-scale demographic pressure and its proximity to terrestrial (evergreen) forest from where the local people collect wood for their daily subsistence needs. Although the two aforementioned mangrove stands are rather less damaged, there is however an urgent need to regulate the anthropogenic pressure in the Cameroon estuary. The development of management strategies should also consider the ecological and economic significance of mangrove products [11,16]. The enforcement of laws that protect mangrove ecosystems, combined with high awareness and alternative job proposals, are determinant for conservation of these coastal ecosystems.

## Competing interests

The authors declare that they have no competing interests.

## Authors' contributions

ANA conducted interviews and field observations, participated in its design, performed the statistical analysis and made interpretation of results and draft the final version of the manuscript. ND participated in the design and final draft of the manuscript. EKGL conducted interviews and field observations. BS and NK participated in the design and final drafts of the manuscript. FDG conceived and supervised the study, participated in its design, made data interpretation and participated in all drafts of the manuscript. All authors read and approved the final version of the manuscript.
